# Metastatic renal cell carcinoma involving colon with unusual histologic features and diagnostic challenges: A case report

**DOI:** 10.1016/j.ijscr.2021.02.013

**Published:** 2021-02-09

**Authors:** Ramin Saadaat, Ahmed Maseh Haidary, Abdul Sami Ibrahimkhil, Jamshid Abdul-Ghafar

**Affiliations:** Department of Pathology and Clinical Laboratory, French Medical Institute for Mothers and Children (FMIC), Kabul, Afghanistan

**Keywords:** RCC, Renal Cell Carcinoma, H&E, Hematoxylin & Eosin, IHC, Immunohistochemistry, GIT, Gastrointestinal Tract, CK, Cytokeratin, APR, Abdominoperineal Resection, EMA, Epithelial Membrane antigen, ASPS, Alveolar Soft Part Sarcoma, Renal cell carcinoma, Metastatic, Colon, Rectum, Rhabdoid features

## Abstract

**Introduction and importance:**

Renal cell carcinoma (RCC) accounts for 3% of all malignancies in adults, on its own being the 3rd most common urologic malignancy. Commonly RCC metastasizes to lung, bone, liver, brain but rarely to colorectum. Here we present the metastasis of RCC to colon with unusual histologic features.

**Case presentation:**

A 40-year-old woman presented with abdominal pain and constipation. Colonoscopy showed an ulcerative mass 30 cm from anal verge. Subsequently, she underwent abdominoperineal resection of the involved portion of colon and the biopsy was sent to us for histopathological analysis. Grossly, it was a large fungating mass. Microscopic examination revealed a malignant neoplasm with polygonal cells, abundant eosinophilic cytoplasm, eccentric nuclei and prominent nucleoli. Immunohistochemistry confirmed the diagnosis of RCC.

**Clinical discussion:**

Colon rarely infiltrated by metastasis from RCC and so far, around 25 of such cases have been reported in the literature. Most common metastatic type of RCC to gastrointestinal tract is clear cell type. In our patient, the type of the metastatic RCC to colon was RCC with rhabdoid features. RCC with rhabdoid features is rare (3–5% of all RCC), but it is highly aggressive with higher chance of metastasis, extra renal invasion and poorer prognosis.

**Conclusion:**

RCC should be considered as one of the differential diagnosis of colorectal cancers. Appropriate immunohistochemical workup would then reveal the correct diagnosis.

## Highlights

• Metastatic RCC in the colon, although very rare, should be kept in the differential diagnosis of patients with lower GI bleeding, particularly patients with a prior history of kidney mass or nephrectomy
• Most common type of primary or metastatic RCC is Clear-cell-type.
• We reported the first case of rhabdoid-RCC metastasizing to colon.
• In the absence of IHC-stains, rhabdoid-RCC may be misdiagnosed as soft-tissue-sarcoma or other poorly differentiated carcinomas.
• Our case exclusively occurred in a woman while colonic metastasis of RCC commonly occurring in males.

## 1 Background

Renal cell carcinoma (RCC) which arises from cortex of the kidney and make up to 85% of renal malignant tumors, is more common in men than women [1]. RCC being the 3rd most common urological cancer, accounts for 3% of all malignancies in adults, following prostate and bladder cancer [2]. The classic initial triad of symptoms that includes hematuria, flank pain and palpable mass, are reported only in up to 17% of patients and the majority of RCC cases are asymptomatic. Due to extensive uses of imaging diagnosis for urological disorders in routine medical practice, majority of the RCC cases are diagnosed incidentally [1].

Generally, RCC is accompanied with 25% events of metastasis and has the potential to metastases to every distant organ after many years. Most common sites where RCCs metastasize are lungs, bones, liver and brain [3]. Metastatic RCC in gastrointestinal tract (GIT) is very rare [3]. Metastatic RCC has poor prognosis with a 5-year survival of 0–18% in patients with untreated metastatic disease [4].

RCC has many different histologic types of which clear cell RCC is the most common one, representing 75–80% of RCC cases [5]. One of the rare but aggressive type of kidney tumor is RCC with rhabdoid features. RCC with rhabdoid features has rapid growth, with up to 70% cases being associated with distant metastasis and ultimately fatal outcome. The median survival rate of RCC is between 8–31 months [6].

Very few cases of metastatic RCC in colon have been reported so far. The first case of metastatic RCC in colon reported in 1991 [7].

Here we present a rare case of RCC with unusual histological features that metastasized to colon. Our work has been reported in concordance with the SCARE criteria [8].

## 2 Case presentation

A 40-year-Old married lady form Kabul presented to the physician with three months’ history of abdominal pain and constipation. No other clinically significant information from the personal, family and drug history was elaborated during the patient interrogation. Colonoscopy was done and showed ulcerative mass at 30 cm of the anal verge. The initial biopsy was done at another hospital and the biopsy specimen was unsatisfactory for evaluation, having no evidence of malignancy. In a private hospital, the patient subsequently underwent a segmental resection of the colon through an Abdominoperineal procedure by a general surgeon with extensive experience in surgical oncology. Accordingly, the specimen was sent to our institution for histopathological evaluation.

### 2.1 Pathology evaluation

**Fig. 1:**
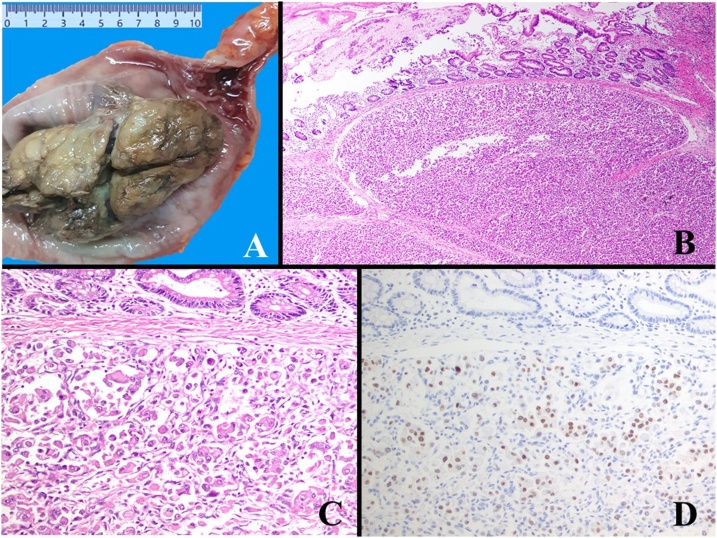
Grossly after opening of the segment of rectum, there was an exophytic mass with lobulated surface (1A). On microscopic low power, a submucosal neoplasm arranged in sheets, alveolar and rhabdoid patterns was seen (1B). The neoplastic cells were pleomorphic having abundant eosinophilic cytoplasm, enlarged hyperchromatic eccentric nuclei and prominent nucleoli (1C). IHC stains were positive for PAX8 (1D).

54 cm segment of colon was received. After opening, it showed an fungating mass with lobulated surface, measuring 11 × 6 × 4 cm in distal portion of the segment (Fig. 1A). Tissue sample from the mass was taken and submitted for microscopic slides examination. On microscopic examination of the sections, a neoplasm arranged in sheets with alveolar and rhabdoid patterns was noted. The neoplastic cells were pleomorphic having abundant eosinophilic cytoplasm, enlarged hyperchromatic eccentric nuclei and prominent nucleoli (Fig. 1B–C). No conventional RCC pattern was seen. Based on examination of haematoxylin and eosin (H&E) stained sections, an impression with differential diagnosis of soft tissue sarcomas, melanoma, large cell neuroendocrine tumor, and poorly differentiated adenocarcinoma was made.

### 2.2 Immunohistochemistry

IHC stains were positive for Cytokeratin (CK) AE1/AE3, Cam 5.2, SATB2, Vimentin and PAX8 (Fig. 1D) and negative for TFE3, Desmin, MyoD1, S100 protein, Anti Smooth Muscle Actin (ASMA), HMB-45, Melan A, CK7, CK20, CDX2, CD34, CD117, Synaptophysin, and Hepar-1. The final diagnosis of metastatic RCC with rhabdoid features was confirmed. Upon further inquiry, the patient revealed history of undergoing a nephrectomy a year ago and the histopathology had been done in another private laboratory. The previous microscopic slides of nephrectomy were requested for review and the diagnosis of RCC with rhabdoid features was also confirmed in the renal mass.

### 2.3 Outcome and follow up

Patient opted for colon Abdominal Perineal Resection, after the surgery, the patient felt well with no symptoms of abdominal discomfort and remained well in the following weeks. Patient didn’t receive chemotherapy after surgery and within the following months her health conditions deteriorated. She passed away 6 months after the diagnosis of metastatic RCC to the colon.

## 3 Discussion

RCC is the most common type of kidney tumor involving individuals between 50–70 year old, more predominant in men, with male to female ratio of 2:1 [9]. As the RCC is a lethal malignancy, its survival mainly depends on the stage at diagnosis, because patient with stage 1 disease have a 5-year survival rate approaching around 93%, while 12% of the patients in stage IV survive beyond 5 years. Other poor prognostic factors are older age at diagnosis, nodal metastases, fat invasion, tumor necrosis and tumor size more than 7 cm. According to available reported data, the estimated 5-year survival rate in the US is 76% and 86.1% in Latin-America [10].

In around 35% of RCC cases, the synchronous metastasis will be present at the time of diagnosis of the primary RCC and about 30% may develop metachronous disease leading to 10% of late diagnosis [11].

Although, RCC rarely metastasize to gastrointestinal tract (GIT) but when it does, it can involve any part of GIT. Metastasis to colon when compared to stomach and small bowel, is rarer [12]. Breast cancers, stomach cancer and malignant melanoma are the most common cancers metastasizing to colon [12]. Metastases can occur through lymphatic, hematogenous or direct invasion. Tumor size is directly proportional to metastatic potential while, metastasis to lymph nodes and distant parts can evenly occur in early stages of RCC [13].

Based on literature review, Kataoka et al., reported that the time from diagnosing from primary tumor to metastasis can occur from months to years (median time 5 yeas). The majority of the cases occurred in males and in older ages. In our case, the metastasis to colon was within the first year, after diagnoses of primary RCC. In our patient, further unique features, occurrence in a woman and was diagnosed when the patient was 45 years of age, while in the previous reports majority of cases were over the age of 60 years and only one case was 35 years of age [14].

According to our literature review, the most common type of RCC is clear cell type, but cases with papillary type, chromophobe type and sarcomatoid type of RCC were also reported [5]. In our patient, the type of the metastatic RCC to colon was RCC with rhabdoid features. RCC with rhabdoid features is rare (3–5% of all RCC), but it is highly aggressive with higher chance of metastasis, extra renal invasion and poorer prognosis [6].

The rhabdoid cells are polygonal with abundant eosinophilic cytoplasm and eccentric nuclei and prominent nucleoli. In IHC the rhabdoid cells are positive for vimentin, Epithelial Membrane antigen (EMA), CK and PAX8 but negative for desmin, myoglobin, myogenin and Myo D1 [15]. To the best of our knowledge, no case of colonic metastasis of RCC with rhabdoid features had yet been reported in the available literature and this patient was the first case. The rhabdoid features should be differentiated from other malignant tumor with same morphology.

Primary alveolar Soft Part Sarcoma (ASPS), although not common in colon, has polygonal cells, having eosinophilic and granular cytoplasm in alveolar pattern. The ASPS is positive for vimentin, Myo D1 and TFE3, our case was negative for Myo D1 and TFE3 [16]. Metastatic pleomorphic rhabdomyosarcoma also should be in consideration as it also has pleomorphic polygonal large rhabdomyoblasts. The cells demonstrate positive immunostaining for myogen, desmin, SMA and vimentin whereas they are negative for RCC with rhabdoid features [17]. In our case the microscopic features were quite similar to ASPS but negative TFE3 and Myo D1 and history of nephrectomy and review of previously nephrectomy slides supported the diagnosis of metastatic RCC with rhabdoid features.

PEComa in colon is very rare but can make challenges in diagnosis for metastatic RCC, as it has polygonal to round cells and clear cytoplasm. Here, again the IHC staining has the leading role for differentiation. PEComa has strong positivity for ASMA and HMB45 stains while RCC is negative [18]. Hence, proper biopsy is the gold standard for diagnosing metastasis and should be done in all situations with adequate precaution. Histopathology with light microscopy alone cannot help much in differentiating RCC metastasis from other clear cell tumors and is challenging [19].

In patients with colonic metastasis of RCC, the overall 5 year survival rates can be less than 10% but surgical resection can improve it up to 88% even the survival improvement can be seen in multi-focal metastasis [20]. Our patient was alive up to six months after diagnosing the metastatic disease.

### 3.1 Conclusion

A colonic neoplasm can be as a result of metastatic RCC, therefore, patients with lower GI bleeding, in particular patients with the past history of kidney mass or nephrectomy should be screened thoroughly by physical examination and imaging modalities. Histologically, different types of sarcomas are having polygonal cells and rhabdoid features. However, our case concluded that RCC should also be considered in tumors with aforesaid histomorphological features, particularly, in the absence of IHC staining facilities.

### 3.2 Patient perspective

The son of the patient stated that it would be beneficial to publish and share this case with other healthcare providers, to enable better understanding and correct diagnosis of such cases (rhabdoid RCC), that would allow provision of specific care and avoidance of its confusion with other neoplasms that display similar morphological features.

## Declaration of competing interest

The authors report no declarations of interest.

## Funding

No financial support was provided for this study.

## Ethical approval

Since this was a retrospective observational study and did not involve actual patients or patient’s images, ethical approval was not sought for this study.

## Consent

Written informed consent was obtained from the patient for publication of this case report and accompanying images. A copy of the written consent is available for review by the Editor-in-Chief of this journal on request.

## Authors contribution

RS and JA-G convinced the idea. JA-G, and RS diagnosis the case. RS was involved in literature review and drafted the manuscript. RS and JA-G helped to collect clinical and follow-up data of the cases; AMH and ASI participated in reviewing the drafted manuscript. JA-G participated with the corresponding, editing the drafted manuscript as per journal policy, and submission of the article. All authors read and approved the final manuscript.

## Registration of research studies

Not applicable.

## Guarantor

Jamshid-Abdul-Ghafar, MD, PhD.

## Provenance and peer review

Not commissioned; externally peer-reviewed.
